# Resolution of Possible Neurosarcoidosis With Early Initiation of Glucocorticoids in the Acute Inpatient Setting: A Case Report

**DOI:** 10.7759/cureus.38686

**Published:** 2023-05-07

**Authors:** Edward J Modica, Jeni E Sacklow, Joseph McCullough

**Affiliations:** 1 College of Osteopathic Medicine, New York Institute of Technology, Old Westbury, USA; 2 Department of Internal Medicine, Division of Hospital Medicine, Northwell Health, Bay Shore, USA

**Keywords:** neuroinflammatory disease, extrapulmonary sarcoidosis, granulomatous disease, sarcoidosis, neurosarcoidosis

## Abstract

Neurosarcoidosis is an autoimmune disorder of unknown etiology. We report a case of a 27-year-old African American male presenting with fever, vomiting, and seizure. Initially, bacterial meningitis was suspected, and empiric antibiotics with dexamethasone were started. Workup revealed negative cultures, leptomeningeal enhancement, and cavitary lung nodules with hilar lymphadenopathy on imaging and elevated angiotensin-converting enzyme levels on cerebrospinal fluid (CSF) analysis. Neurosarcoidosis was then suspected, and a lung biopsy was performed. The results were inconclusive, but the patient’s condition improved. He was discharged on prednisone. Our case demonstrates the diagnostic difficulty of neurosarcoidosis while displaying the importance of early initiation of glucocorticoids in the acute inpatient setting.

## Introduction

Sarcoidosis is an immune-mediated disorder of unknown etiology, characterized by granulomatous inflammation of multiple organ systems [1-7]. Pathologically, it is defined by the presence of noncaseating granulomas [1-7]. Neurological involvement or neurosarcoidosis is a less common form but can affect any part of the nervous system [1-7]. It has been reported in two per 100,000 of the general population and 5%-26% of all patients with sarcoidosis [1-3,5-7]. The diagnosis of neurosarcoidosis is difficult, with pathological evaluation as the main determinant between the three diagnostic categories: definite, probable, and possible [1,4,7]. To make a definite diagnosis of neurosarcoidosis, confirmed evidence of sarcoidosis on central nervous system (CNS) pathology is necessary. A probable diagnosis requires confirmed systemic pathology. A possible diagnosis of neurosarcoidosis can be made if there is no pathologic evidence of sarcoidosis [1,4]. The mainstay of treatment for neurosarcoidosis is glucocorticoids [1-3,5,7]. Second- and third-line therapies include hydroxychloroquine, azathioprine, cyclophosphamide, methotrexate, infliximab, and adalimumab, but data is limited [1-3,5,7]. The prognosis of neurosarcoidosis is largely based on case studies and cohort studies, with one meta-analysis finding total remission in 27%, incomplete remission in 32%, stable disease in 24%, and deterioration in 6%. Mortality was reported in a range between 0% and 33% [7]. The 10-year survival rate has been reported around 89% with a 10-year relapse rate of 86.2% [8].

## Case presentation

Patient information

A 27-year-old African American male presented to the emergency department with nausea, vomiting, and a severe headache for 12 hours. His headache began insidiously the night prior, but due to worsening pain, he drove himself to the emergency department. During triage, the patient experienced expressive aphasia and would only respond with head nodding. Shortly after this, the patient was witnessed having a tonic-clonic seizure. On further investigation, the patient’s girlfriend provided a history that included chills, night sweats, and unintended weight loss for six months leading up to the patient’s current presentation.

This patient had a history of legal incarceration occurring two years ago and a past medical history of childhood asthma. The patient reported that he frequently smoked marijuana but denied the use of other drugs, tobacco, or alcohol. He was not familiar with tuberculosis and thus has no knowledge of exposures. Further history from his family revealed a family history of thyroid disease in his maternal grandmother but denied any autoimmune conditions, including sarcoidosis, systemic lupus erythematosus, or rheumatoid arthritis.

The patient had previously been to the emergency department three times in the four months leading up to his current presentation. Each encounter was secondary to complaints of nausea and vomiting, which were resolved with fluids and metoclopramide.

Clinical findings

Directly after the patient’s witnessed seizure, abortive therapy was attempted with a 2 mg intravenous (IV) push of lorazepam without success. A second attempt with a 2 mg IV push of midazolam was successful, and the patient was intubated for airway protection with concern for status epilepticus. Initial vitals signs showed a temperature of 100.6°F, heart rate of 88 BPM, blood pressure of 125/72 mmHg, respiration rate of 23, and SpO2 of 100% on room air. On physical examination, the patient was intubated and sedated to a Richmond Agitation Sedation Scale (RASS) of -5. Cranial nerve examination revealed midline gaze, 2 mm pupils, and reactive to light. Lower extremities had positive clonus bilaterally, along with spontaneous contractions in bilateral upper extremities. No rashes or skin changes were noted. Physical examination was limited due to sedation but did not reveal any other abnormalities of the cardiovascular, pulmonary, gastrointestinal, or vascular systems.

Diagnostic assessment

**Figure 1:**
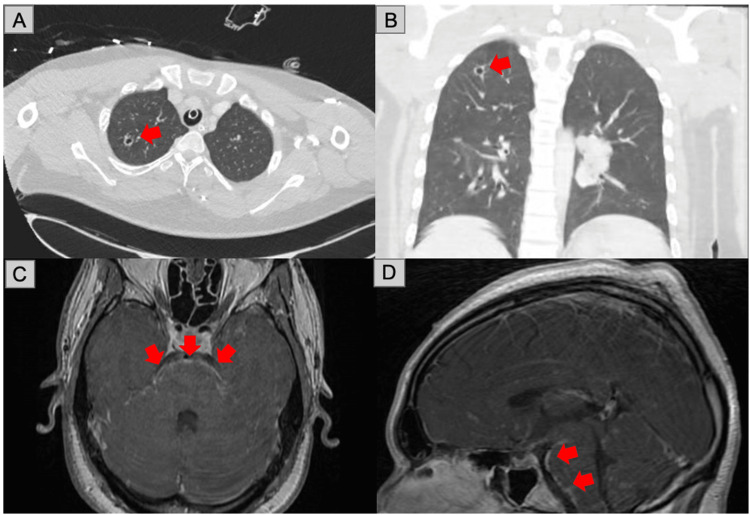
Radiologic evaluation (A) Axial CT of the chest with contrast (arrow). (B) Coronal CT of the chest with contrast: mediastinal and bilateral hilar lymphadenopathy; cavitary and spiculated nodules in the right upper and middle lobes, the largest measuring 1.2 cm in the right upper lobe (arrow). (C) Axial T1-weighted MRI with contrast (arrows). (D) Sagittal T1-weighted MRI with contrast: abnormal leptomeningeal enhancement along the surface of the brainstem (arrows). CT: computed tomography, MRI: magnetic resonance imaging

Initial laboratory findings included a complete blood count with differential, comprehensive metabolic panel, and thyroid function tests that were unremarkable. Electroencephalogram (EEG) was performed for 15 hours continuously while the patient was sedated and revealed severe nonspecific diffuse or multifocal cerebral dysfunction with no epileptic activity. Following this, a computed tomography angiography (CTA) of the head and neck with Omnipaque 350 mg/mL contrast revealed severe/critical narrowing of the right distal M1 through proximal M2 branches as well as an incidentally noted 1.5-cm thick-walled cavitary lesion in the right lung apex and additional scattered nodules. Due to the patient’s history and presentation, a lumbar puncture was performed on hospital day 2 and revealed low glucose, elevated protein, elevated neutrophil count, and increased opening pressure (Table 1). Magnetic resonance imaging (MRI) of the brain with gadolinium contrast revealed leptomeningeal enhancement, greatest in the posterior fossa, without focal brain parenchymal abnormality and no enhancement with contrast (Figure 1). Cerebrospinal fluid (CSF) polymerase chain reaction (PCR) was negative with increasing suspicion of tuberculous meningitis.

**Table 1:** Laboratory evaluation CSF: cerebrospinal fluid, ACE: angiotensin-converting enzyme, SARS-CoV-2: severe acute respiratory syndrome coronavirus 2, P-ANCA: perinuclear antineutrophil cytoplasmic antibody, C-ANCA: cytoplasmic antineutrophil cytoplasmic antibody, ANA: antinuclear antigen

CSF	Value	Normal range
Protein	226 mg/dL	15-45 mg/dL
Glucose	15 mg/dL	40-70 mg/dL
Total nucleated cell count	17/uL	0-5/uL
Appearance	Clear	Clear
Red blood cell count	83/ccm	0-1/ccm
Lymphocytes	60%	40%-80%
Monocytes/macrophages	18%	15%-45%
Neutrophils	22%	0%-6%
Organisms	None	None
Culture	No growth	No growth
Gram stain	Negative	Negative
ACE	10.9 mg/dL	0-2.5 mg/dL
PCR: *Escherichia coli* K1, *Haemophilus influenzae*, *Listeria monocytogenes*, *Neisseria meningitidis* (encapsulated), *Streptococcus pneumoniae*, *Streptococcus agalactiae*, *Cytomegalovirus*, SARS-CoV-2, *Enterovirus*, herpes simplex virus-1, herpes simplex virus-2, human herpes virus-6, human parechovirus, varicella-zoster virus, and *Cryptococcus neoformans*/*Cryptococcus gattii*	Not detected	Not detected
Rheumatologic laboratory values	Value	Normal range
C-reactive protein	112 mg/L	≤4 mg/L
P-ANCA	Negative	Negative
C-ANCA	Negative	Negative
ANA	Negative	Negative
Tuberculosis workup	Value	Normal range
Acid-fast bacteria sputum smear	Negative	Negative
Acid-fast bacteria CSF smear	Negative	Negative
QuantiFERON-TB Gold	Negative	Negative

Due to the patient’s persistent febrile state despite the initiation of antimycobacterial therapy detailed below, further workup was continued for infectious and autoimmune sources. Thirteen acid-fast bacteria smears from various sources were negative, and QuantiFERON-TB Gold as well as cultures for fungal or bacterial origin were consistently negative. CSF cultures continued to have no growth on day 6 (Table 1). Repeat EEG showed severe generalized background slowing and no epileptiform activity unchanged from the previous study. Chest computed tomography (CT) without contrast revealed an irregular 1.2-cm lateral right middle lobe nodule and additional small cavitary and solid nodules at the right apex as well as noncalcified mediastinal and hilar lymphadenopathy (Figure 1). Despite a CSF glucose (15 mg/dL) of less than two-thirds of the serum glucose (88 mg/dL), negative gram stains of CSF, improving clinical condition, and normalization of serum leukocyte count, there was a low suspicion for bacterial meningitis. Due to high suspicion of sarcoidosis based on chest CT findings and positive angiotensin-converting enzyme (ACE) level in CSF, the patient underwent endobronchial ultrasound with biopsy (EBUS) for sarcoidosis workup. The EBUS results were nondiagnostic, revealing benign bronchial cells and pulmonary macrophages in a background of mucin and red blood cells, and no granulomas or carcinoma were identified in any of the specimens due to non-representative sampling.

Therapeutic intervention

After the administration of the lorazepam and midazolam as stated above, the patient remained sedated with propofol. He was additionally treated with levetiracetam 1 g every 12 hours for seizure prophylaxis. Following his lumbar puncture, he was started on empiric antibiotic therapy with intravenous vancomycin 2,000 mg every 12 hours and intravenous ceftriaxone 2,000 mg every 24 hours. This was initiated along with IV dexamethasone 10 mg every 24 hours. This continued from hospital day 2 to hospital day 5 when the antibiotic regimen was expanded. After continued workup and increasing suspicion of tuberculous meningitis, the patient was started on antimycobacterial therapy with rifampin, isoniazid, pyrazinamide, and ethambutol (RIPE) on hospital day 3. By hospital day 8, acid-fast smears, CSF gram stains, and PCR were confirmed negative, and all antibiotic therapy was ceased. Based on the increasing suspicion of sarcoidosis or another autoimmune cause, the patient underwent endobronchial ultrasound with biopsy (EBUS) for a sarcoidosis workup. The patient was transitioned to oral prednisone 50 mg two times a day as well as levetiracetam 500 mg daily. He continued to have good clinical improvement at this time with no further seizures. The patient was ambulating unassisted with no complaints of headache or dizziness.

Follow-up and outcomes

Following the patient’s consistently negative bacterial cultures and continued clinical improvement, he was deemed safe and stable for discharge from the hospital on day 9. Following discharge, he followed up with rheumatology and neurology for this presumed diagnosis of neurosarcoidosis despite the inconclusive biopsy. The patient’s steroid dose was then decreased from 50 mg twice daily to 40 mg once daily, and he was maintained on levetiracetam 500 mg daily. He continued to experience intermittent headaches, nausea, and tremors. The patient was then scheduled to obtain a positron emission tomography (PET) scan and follow up for outpatient visits in 4-6 months. However, the patient was brought to the emergency department a month and a half following the initial admission for unwitnessed seizure activity, despite reported adherence to his medical regimen. Upon this second seizure and admission, the patient’s steroid dose was increased to 60 mg daily pending further imaging with PET and CT scans.

**Table 2:** Timeline of events CTA: computed tomography angiogram, ACE: angiotensin-converting enzyme, MRI: magnetic resonance imaging, RIPE: rifampin, isoniazid, pyrazinamide, and ethambutol, CSF: cerebrospinal fluid, PCR: polymerase chain reaction, EBUS: endobronchial ultrasound

Hospital day	Events
1	The patient presented to the emergency department, followed shortly after by a tonic-clonic seizure, and was intubated following abortive therapy. CTA of the head and neck revealed a 1.5-cm thick-walled cavitary lesion in the right lung apex.
2	The patient was intubated/sedated. Lumbar puncture suggestive of bacterial meningitis with positive ACE. Broad-spectrum antibiotics and dexamethasone were initiated. MRI of the brain was obtained revealing leptomeningeal enhancement. Infectious workup continued.
3	Concern for tuberculosis, RIPE therapy added. Continued infectious workup.
4	CSF gram stain negative. Continue broad spectrum and RIPE antibiotics. Infectious workup pending.
5	Acid-fast smear negative ×4. Antibiotics continued. Pending CSF PCR.
6	Begin weaning sedation. Antibiotics continued. CSF PCR pending.
7	CSF PCR and cultures negative. Sputum acid-fast bacillus negative ×6. Dexamethasone continued. High suspicion for sarcoidosis with the current clinical picture.
8	The patient was extubated. All antibiotics were discontinued due to low suspicion of infectious cause. Autoimmune workup continued.
9	On steroids and clinically improving. EBUS with biopsy performed. Discharged with levetiracetam and prednisone.
3 weeks post-discharge	Outpatient rheumatology follow-up appointment. Prednisone dose decreased.
6 weeks post-discharge	Returns to the emergency department with reported seizure activity. Prednisone dose was increased, and he was discharged.

## Discussion

Sarcoidosis most commonly affects the lung, skin, eyes, liver, and lymph nodes [1-3,5-7]. The incidence in African Americans is reported to be higher in the United States at 35-80 per 100,000 [1]. Our patient, being African American with a slightly higher risk for sarcoidosis, presented with lung and neurological involvement.

Neurosarcoidosis, while rare, was found to be the first manifestation of disease in 52% of patients diagnosed with sarcoidosis [6], which is similar to our patient. Prior to this, he did notice systemic symptoms, but his severe headache, nausea, and seizure on arrival are what prompted a further workup. CNS manifestations are more common (80%) than peripheral nervous system (PNS) manifestations [6], with it most commonly presenting as cranial neuropathies (50%-75%) [1,4,6,7]. Our patient presented with signs and symptoms of CNS inflammation, likely indicated by the leptomeningeal enhancement seen on MRI. Aseptic meningitis accounts for 10%-20% [1,4,6,7] of neurological manifestations, with leptomeningeal involvement seen in approximately 40% of patients on MRI [6].

Due to the extensive workup, it was not until hospital day 7 that neurosarcoidosis became top of our differential. CSF ACE levels have low sensitivity and specificity for neurosarcoidosis [1]. Our patient’s CSF findings were consistent with a previous study, which found that a low glucose level with an elevated ACE level in the CSF was exclusive to neurosarcoidosis [1,9]. Following a comprehensive assessment of alternative etiologies (malignancy and infection), the patient’s clinical manifestations (fever, unintended weight loss, etc.), radiological findings, and elevated ACE levels in the CSF all raised suspicion for neurosarcoidosis. The 2018 Neurosarcoidosis Consortium Consensus Group differentiates diagnosis into three categories: definite, probable, and possible [1,4]. Due to the indeterminate EBUS pathology findings, the patient’s presentation met the criteria for a possible diagnosis.

Glucocorticoids are first-line therapy [1-3,5,7]. Early initiation of treatment can halt the progression of the disease and avoid permanent neurological deficits [1]. Our patient was started on dexamethasone on hospital day 3 due to concerns about bacterial meningitis. It was continued throughout his hospital course as a diagnosis of neurosarcoidosis became more likely. By hospital day 9, he had regained his mental status and functional capacity and was stable for discharge. This robust response to glucocorticoid therapy supports a diagnosis of neurosarcoidosis.

Over 80% of patients with neurosarcoidosis relapse, and patients with encephalic involvement are at increased risk [8]. Due to our patient’s encephalic involvement, he was at a higher risk for relapse, which did occur almost two months after discharge with breakthrough seizure activity. If his symptoms continue to occur on steroid therapy, a switch to second- and third-line therapies may be warranted. Close follow-up and further work-up are needed in the outpatient setting to elucidate the exact etiology of his illness and prevent additional sequelae.

## Conclusions

Prompt workup and the start of therapy are important to avoid potentially permanent neurological deficits. Fortunately for our patient, glucocorticoids were started early, and further neurological deficit was avoided. His initial condition improved without the need for second- and third-line therapies. It is difficult to say that neurosarcoidosis should be high on any physician’s differential, but when the possibility is identified, initiating glucocorticoid therapy may provide some benefit in the acute inpatient setting, as demonstrated by our case.
